# Hydrophilic Interaction Liquid Chromatography-Tandem Mass Spectrometry Analysis of Fosetyl-Aluminum in Airborne Particulate Matter

**DOI:** 10.1155/2018/8792085

**Published:** 2018-03-01

**Authors:** Francesca Buiarelli, Patrizia Di Filippo, Carmela Riccardi, Donatella Pomata, Riccardo Marsiglia, Carla Console, Daniele Puri

**Affiliations:** ^1^Department of Chemistry, University “Sapienza”, Piazzale Aldo Moro, 5-0185 Rome, Italy; ^2^Inail DIT, Via Roberto Ferruzzi, 38-00143 Rome, Italy; ^3^DIT, INAIL, Via di Fontana Candida, 1-00040 Monteporzio Catone, Rome, Italy

## Abstract

Fosetyl-aluminum is a synthetic fungicide administered to plants especially to prevent diseases caused by the members of the Peronosporales and several *Phytophthora* species. Herein, we present a selective liquid chromatography-tandem mass spectrometry (LC-MS/MS) method to analyze residues of fosetyl-A1 in air particulate matter. This study was performed in perspective of an exposure assessment of this substance of health concern in environments where high levels of fosetly-Al, relatively to airborne particulate matter, can be found after spraying it. The cleanup procedure of the analyte, from sampled filters of atmospheric particulate matter, was optimized using a Strata X solid-phase extraction cartridge, after accelerated extraction by using water. The chromatographic separation was achieved using a polymeric column based on hydrophilic interaction in step elution with water/acetonitrile, whereas the mass spectrometric detection was performed in negative electrospray ionization. The proposed method resulted to be a simple, fast, and suitable method for confirmation purposes.

## 1. Introduction

Fosetyl-Al is a broad-spectrum fungicide, rapidly absorbed by leaves and roots of various horticultural crops, that acts by blocking or inhibiting the germination of spores, the development of mycelium, and the penetration of the pathogens into the plants.

It is worldwide used to control a range of diseases caused by different species such as *Phytophtora*, *Plasmopara,* and *Phytium*. Modes of application are different and can include root, soil, foliar, trunk, and seed treatment [1, 2]. Since 1977, fosetyl-Al was recognized to exhibit antifungal activity, despite not having explicit awareness of the site of action in target pathogens. Recently however, it was reported that efficacy is based on an indirect action through stimulation of the natural plant defenses. It is found as wettable dispersible granules or wettable powder [3, 4], and sometimes it is in coformulated mixtures with other pesticides.

Fosetyl-Al, aluminum tris(*O*-ethyl phosphonate), is the aluminum salt of the phoshonic acid monoethyl ester. It has a molecular mass of 354 Da, and it is highly polar and ionic, soluble in water, and poorly soluble in most common organic solvents except methanol. When dissolved in water, it rapidly degrades to phosphonic acid. It metabolizes to ethanol and phosphorous acid [5]. The toxicological profile of fosetyl-Al is reported on the FAO Evaluation Report 2013 [3].

From the analytical point of view, the analysis of fosetyl by gas chromatography (GC) needs derivatization for the conversion of ethyl phosphonate into a volatile methyl ester [6]. In liquid chromatography (LC), fosetyl was analyzed by high-performance ion chromatography (HPIC) using two columns, respectively, CarboPac and IonPac; the latter used specifically for the analysis of organic anions and acids [7]. HPLC analysis was carried out mainly with column C_18_ [8] but also by hydrophilic interaction liquid chromatography (HILIC) [9, 10]. A cyan column was used in liquid chromatography with supercritical fluid (SFC) [11].

In a previous paper [12], we simultaneously determined nine pesticides by HPLC-tandem mass spectrometry (MS/MS) in airborne particulate matter of aerodynamic diameter ≤4 *µ*m (PM_4_). In particular, airborne pesticide concentrations were measured inside/outside tractor cabins during the spreading to assess the safety level of the vehicle.

In this paper, we focused on the analysis of fosetyl that was not considered in the previous work [12]. In detail, after choosing the best chromatographic and mass spectrometric conditions for the analysis, we optimized the extraction and purification of the analyte both from a mixture sprayed in an agricultural field and from PM_4_ collected inside and outside the cabin of a tractor engaged in the spraying operations.

Since it is not easily analyzed by reverse-phase liquid chromatography, it showed a good retention by the ZIC®-pHILIC column with a step elution. Mass spectrometry operated in negative electrospray ionization (ESI) in multiple reaction monitoring (MRM) mode. The sample preparation was based on an accelerated solvent extraction of the analyte from the commercial mixture or from sampled filters, followed by purification on solid phase before the analysis by liquid chromatography. Water, the greenest solvent [13], was used to extract and purify the analyte.

Results obtained on the spread mixture showed that the method is accurate and precise, whereas results obtained on environmental samples highlighted the efficacy of the tractor cabin used in the sampling sites.

In addition, considering the solid nature of the environmental samples (filters), this method could be reasonably applied to other kinds of samples such as food or soil.

## 2. Materials and Methods

### 2.1. Chemicals, Reagents, and Materials

Fosetyl-aluminum (technical grade) was purchased from Sigma-Aldrich S.r.l. (Milano, Italy).

Individual stock standard was prepared at 1 mg·ml^−1^ in water and stored in the dark at −20°C. Working standard solutions were prepared by diluting the stock standard and kept at +4°C in amber vials.

The mixture dispensed on agricultural field was R6 ERRESEI ALBIS™ (Bayer CropScience AG, Dormagen, Germany), containing fosetyl at 66.7% [14].

Solvents such as methanol (MeOH) and acetonitrile (CH_3_CN) (ultra gradient, ultra purity solvent) were purchased from Sigma-Aldrich S.r.l. (Milano, Italy); water ultra HPLC grade was from ROMIL (Cambridge, GB).

Materials such as diatomaceous earth sorbent (hydromatrix) was obtained from Dionex (Thermo Fisher Scientific, Sunnyvale, CA); polytetrafluoroethylene (PTFE) membrane disc filters (diameter: 37 mm, porosity: 2.0 *µ*m) were from Pall Corporation (VWR International S.r.l., Milan, Italy); Strata X (200 mg/6 ml) cartridges, and Luna C_18_ column (150 mm × 2 mm, 5 *µ*m) were purchased from Phenomenex (Torrance, CA, USA); and SeQuant ZIC-pHILIC polymeric column (150 × 2.1 mm, 5 *µ*m) was acquired from Merck S.p.a (Vimodrone, Milano, Italy).

### 2.2. Sampling

Sampling campaign was performed and realized according to our previous paper [12] in summer 2016 in a vineyard for a total time of 150 min. The sprayed mixture contained fosetyl at a concentration of 1700 mg·L^−1^. 9 kg of R6 ERRESEI ALBIS was dissolved in 3600 L of water and dispensed on the agricultural field. In order to assess the extent and degree of worker exposure, the respirable fraction of particle matter (PM_4_) [15] was collected inside/outside a tractor cabin by filtration with Personal Air Samplers Low Volume (Pumps SKC DeLuxeModel 224-PCX-R8, AMS Analitica, Pesaro, Italy), operating at 0.15 m^3^·h^−1^ (2.5 L·min^−1^), with cyclones for the separation of respirable fraction of particulate matter. The total sampled air volume was about 0.4 m^3^. Field blank filters were placed both inside and outside the cabin. Filters were weighed before and after sampling on an analytical balance (Sartorius MC-5, Δ*m* = ±0.001 mg), after conditioning for twenty-four hours in a chamber maintained at 50% relative humidity and 20°C (Activa Climatic Cabinet, Aquaria, Milano, Italy), in order to determine PM_4_ concentration in air. All filters were sealed and stored in aluminum foils at −20°C. The filters were then processed following the procedure illustrated in Section 2.3.

### 2.3. Sample Preparation

**Figure 1:**
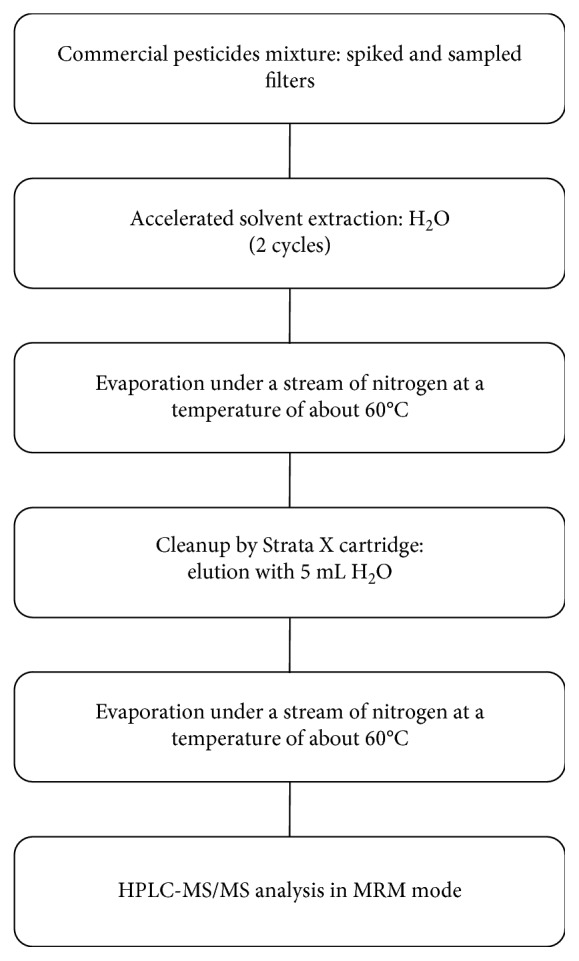
Block diagram of the whole analytical procedure for the analysis of fosetyl from environmental samples, spiked filters, and commercial pesticide mixture.

The preparation of the sample of sprayed mixture and of the sampled filters was carried out according to the procedure described in Figure 1. The sample extraction and purification of the extracted solution is similar to the one described in detail in Di Filippo et al. [12]. After the gravimetric determination of particle mass concentration, extraction was carried out with two successive cycles by ASE Dionex (ThermoFisher Scientific, Sunnyvale, CA) with H_2_O. After evaporation, a solid-phase cleanup was performed by applying the extracts to Strata X cartridges and eluting the analyte immediately with 5 mL of H_2_O. The eluate was dried under nitrogen stream and redissolved in 100 *µ*L of water.

### 2.4. LC-MS/MS Equipment and Conditions

**Figure 2:**
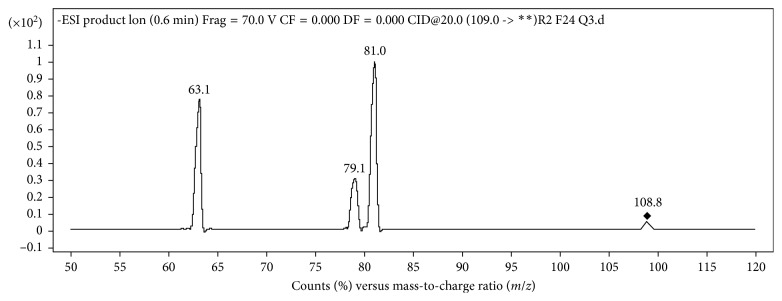
MS/MS spectrum in negative ESI. Infusion (10 *µ*l·min^−1^) of fosetyl aqueous solution at 10 mg·L^−1^. Electrical parameters are as in Table 1. Precursor ion *m*/*z* 109.

A 1290 Infinity HPLC pump system (Agilent Technologies, Santa Clara, CA, USA) with an Agilent G4226A autosampler was coupled to an Agilent G6460 triple quadrupole mass spectrometer equipped with the electrospray jet stream interface (ESI). In order to perform MS and MS/MS analyses in full scan (mass range *m*/*z* 50–500) and in product ion mode, the acquisition parameters were optimized by infusion at a flow rate of 10 *μ*L·min^−1^ of a solution of fosetyl in water without additives (10 ng·*μ*L^−1^). Nitrogen was used as a nebulizing and collisional gas. The fragmentor potential was optimized in order to maximize the parent ion intensities, and, by operating in product scan mode, the collision energy (CE) was optimized (Table 1). Finally, all the analyses were carried out by LC-MS/MS in MRM mode, acquiring diagnostic product ions from the chosen precursor to obtain high specificity and sensitivity. Three main fragments 81, 63, and 79 *m*/*z* were formed from the precursor *m*/*z* 109; the ion *m*/*z* 81 was chosen as a quantifier for the definitive MRM analyses. MassHunter Software was used for the acquisition and the elaboration of the data set.  Figure 2 shows MS/MS spectrum obtained with the electrical parameters in Table 1.

### 2.5. Calibration Curves for Quantitative Analysis and Matrix Effect

Two calibration curves were built in HPLC-MS/MS, in MRM mode. Curve “A” was built using five standard solutions with increasing analyte concentrations (1, 5, 10, 50, 100, 300, 500, and 700 ng·mL^−1^) to evaluate the instrumental linearity. Matrix-matched calibration curve “B” was prepared to evaluate the method linearity and to estimate any possible matrix effect. To mime the environment in which the analytes are found and the interactions between the analytes and other compounds in the matrix (possibly altering the analytical response), nine filters were sampled at the Botanical Garden of the University “La Sapienza” and eight of them were spiked with the same standard solutions of curve “A” and processed, according to the analytical procedure of Figure 1, prior to the injection. The eventual endogenous contribution was subtracted from the analyte response. A linear plot of the peak analyte area against the amount of standard added (abscissa) was drawn. Each solution was injected three times, and the regression model was applied to the calibration data set. The matrix effect was determined by the ratio (*B*/*A* × 100) between the slope of the curve (*B*) and the slope of the standard calibration curve (*A*). A value >100% corresponds to a signal enhancement, whereas a value <100% to a signal suppression [16].

### 2.6. Limit of Detection and Quantification

Limit of detection (LOD) of the method was determined by spiking blank filters with the analyte before the whole procedure. The concentration of the injected analyte producing a peak with a signal-to-noise ratio (S/N) of 3 was chosen as LOD. The limit of quantification (LOQ) was estimated, in the same way as the LOD, using the criterion (*S*/*N*) of 10.

### 2.7. Reproducibility and Precision

Intraday and interday reproducibility of the method was determined by repeating, ten times, the analysis of blank filters spiked at LOQ level, during the same day and five nonconsecutive days and was expressed as relative standard deviation (RSD). The precision of the investigated method was assessed via replicate analyses of the solution coming from the filters spiked by the sprayed mixture.

### 2.8. Recovery and Accuracy

Total recovery was determined on spiked blank filters, before the extraction, with 1 (LOQ level), 1.5, and 2 ng·mL^−1^. The solutions were analyzed in triplicate by LC-MS/MS in MRM mode. The accuracy of the method was assessed via replicate analyses of the solution coming from the filters spiked by the sprayed mixture.

## 3. Results and Discussion

### 3.1. Optimization of HPLC-MS/MS

Firstly, the mass spectrometer parameters were optimized for the analyte to determine suitable source parameters for the best sensitivity and *S*/*N* ratio and to study the fragmentation.  Figure 2 shows the MS/MS spectrum obtained with the electrical parameters in Table 1. The precursor ion (*m*/*z* 109) corresponds to [C_2_H_6_PO_3_]^−^. The loss of acetylene from the precursor, due to a McLafferty rearrangement, provides the ions *m*/*z* 81 and 63 [9]. The transition 109 → 81 was the most intense and therefore used for quantification purposes, whereas the others are used as qualifier ions.

**Figure 3:**
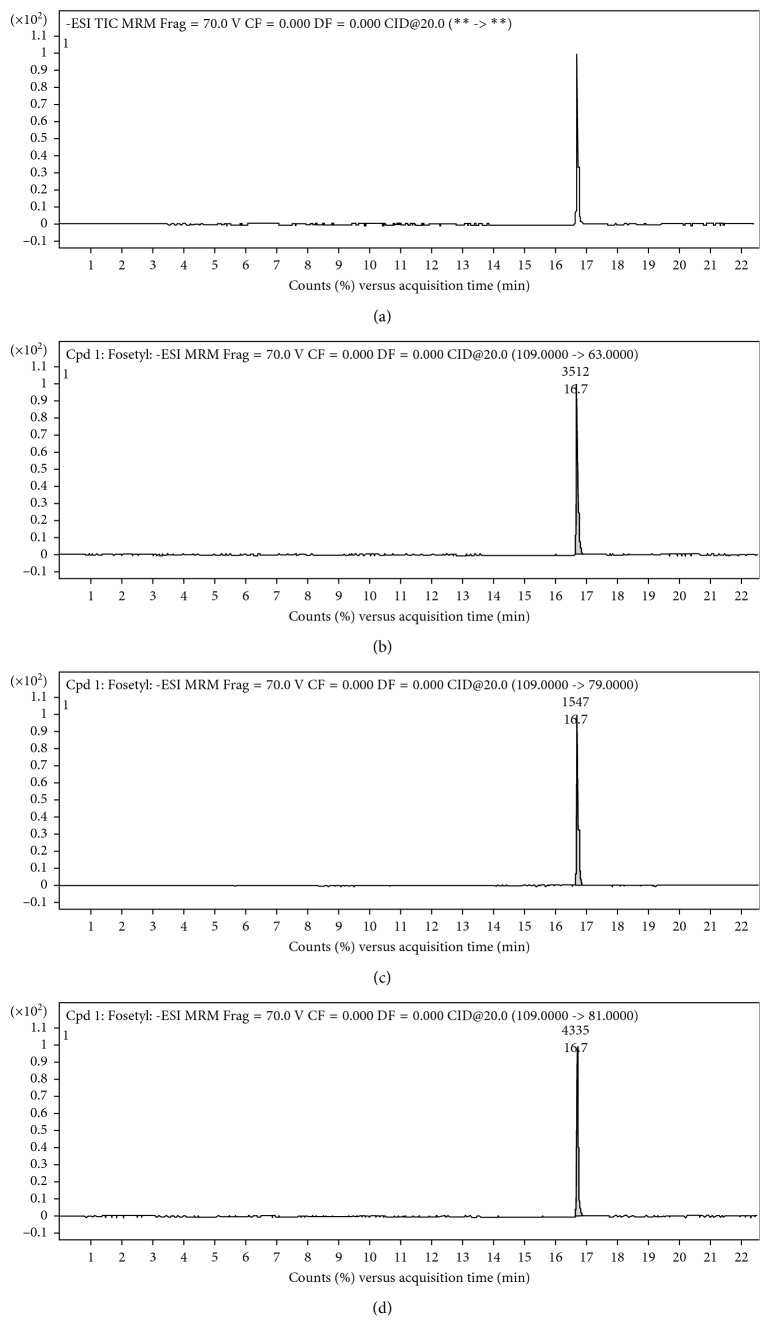
HPLC-MS/MS in the MRM mode of a standard solution of fosetyl. The first window shows the TIC, the second and third windows show the qualifier transitions (*m*/*z* 109 → 63 and *m*/*z* 109 → 79), and the fourth window shows the quantifier transition (*m*/*z* 109 → 81). Chromatographic and mass spectrometric conditions are as in Section 3.1 and in Table 1.

The chemical nature of the investigated analyte has complicated the choice of the chromatographic column to detect the compound. Fosetyl is very hydrophilic, and two columns with different stationary phases, length, and diameters were tried in the following order: C_18_ and ZIC-pHILIC. The different columns showed, as expected, different selectivities. C_18_, despite the presence of a counterion in the mobile phase, did not properly retain the analyte (data not shown) that was eluted with the dead volume. On the other hand, the stationary polymeric porous phase of the ZIC-pHILIC binds covalently zwitterionic groups of sulphobetaine type CH_2_–CH_2_–CH_2_–SO_3_^−^. The hydrophilic and permanent zwitterionic feature makes the column suitable for the retention of poorly retarded analytes, such as fosetyl, in the reversed phase columns, thanks to the weak electrostatic interactions. A step elution, changing sharply (after 8′) the mobile-phase composition from H_2_O-CH_3_CN = 3:97 to H_2_O-CH_3_CN = 50:50 was used to perform the analysis at a working flow rate of 100 *µ*L·min^−1^. Under these conditions, the compound was eluted in 16.5 min as a sharp peak. The oven was maintained at 40°C, and the column was used according to the manufacturer's instruction. The MRM chromatogram of a standard solution of the analyte is reported in Figure 3. The first window shows the total ion chromatogram (TIC), and the others show the extract ion chromatograms. The reproducibility of the retention time was ±0.5%. The retention time of about 17 min allowed us to analyze environmental samples where some interferences are eluted in the first part of the chromatogram (data not shown).

### 3.2. Sample Preparation and Recovery

According to EUPT-SRM 8 April/May 2013 [17], analyses have to be performed in a short period of time to avoid degradation of fosetyl and by following all the precautions for the storage.

As for the sample preparation of Figure 1, both in ASE and in solid-phase extraction (SPE), no organic solvents were used to extract and elute the compound of interest. In fact, water proved to be the most efficient solvent, meeting the requirements of sustainable chemistry, being the greenest solvent.

The recovery was measured on blank filters spiked by three different concentrations of the analyte and submitted to the whole procedure. The recovery was determined as *R* = *C*/*C*_ref_ × 100, where *C* is the concentration found with the method and *C*_ref_ is the reference (added) concentration. The results show that recovery was not dependent on the level of the added concentration. The total recoveries, expressed as the average of three different samples, were always above 80%, with a CV below 20%. Since the extraction by ASE gave a recovery of 100% and the loss due to the evaporation was less than 5%, we supposed that most of the analyte losses were due to the SPE step (data not shown). In this protocol, the solid phase is simply used to “filter” the sample. The cartridge retained the interfering components since analyte molecules showed no interaction with the adsorbent.

### 3.3. Linearity, LOD/LOQ, Reproducibility, and the Matrix Effect

Good linearity was obtained in the investigated concentration range as demonstrated by *R*^2^ value of 0.999 of the calibration curve (*A*) (*y* = 2644*x* + 14067). The matrix effect, calculated as in Section 2.5, was negligible (<10%); therefore, curve A was used for the quantification of environmental samples, and the results were corrected by the recovery.

Table 2 shows LOD and LOQ values for fosetyl expressed as ng·mL^−1^ and as pg·m^−3^ (considering a sampled air volume of 0.4 m^3^) and as mg·kg^−1^ of particulate matter (considering a sampled average particulate matter of 60 *μ*g/filter).

As for LOD, other authors found, in food matrices [18], comparable values (0.2 mg·kg^−1^) of fosetyl. In air particulate matter (PM_2.5_), Coscollà et al. [19] found, for other pesticides by LC-MS/MS, LOQ values with the same order of magnitude (pg·m^−3^). The intraday reproducibility and interday reproducibility at the LOQ level were, respectively, 6% and 10%.

### 3.4. Accuracy and Application of the Sprayed Mixture

The sprayed mixture had a fosetyl concentration of 1700 mg·L^−1^. Three aliquots of 50 *µ*L of mixture (containing 85 *µ*g of fosetyl) were added to three blank filters and subjected to the whole procedure of Figure 1. After SPE, the eluate was dried and diluted to 0.5 L of water, before the chromatographic injection, to be inside the calibration curve. Analyses were repeated three times and quantified on the standard calibration curve. The average of the results (corrected by the recovery) was 1593 mg·L^−1^ with a precision calculated as coefficient of variation (CV) below 10%. The accuracy was calculated as % difference between the experimental and the nominal sample concentrations. The value of 6.3% is more than acceptable for complex analytical methods.

Finally, we compared our results to those currently available for fosetyl-Al residues. To this aim, in Table 3, the performances of this methodology compared to those obtained in food are reported.

As shown, despite the different sources of the samples and the different sample manipulations, the performance of this method is comparable to other validated methods.

### 3.5. Application of Environmental Samples

Environmental samples were collected during a summer campaign (June 2016) in an agricultural site nearby Rome. Four samples of PM_4_ were collected for a sampling time of 2.5 hours, two inside and two outside the tractor cabin, during the pesticide spraying operation. Filters were previously weighed to determine PM_4_ concentration in the air. The particulate matter amount on each filter ranged from 50 to 70 *µ*g. After conditioning, filters were processed, according to the procedure in Figure 1. During the sampling campaign, two field blank filters were also used as controls and exposed to the atmosphere passively and then processed and analyzed.

The results of the sampling campaign in summer 2016 showed an amount of fosetyl, extracted from the sampled particulate matter inside the cabin, lower than LOD. This result demonstrated the effectiveness of the tractor cabin and the safety for the operator. As expected, small amounts were found outside the tractor (98.5 ± 1 ng), whereas blank values were below the LOD inside the cabin and 7.5 ± 0.5 ng outside the cabin.

Having found small amounts of the pesticide on the sampled filters, despite the high concentration in the sprayed mixture, it was interesting to also analyze the particulate fraction with aerodynamic diameter higher than 4 *µ*m (inhalable fraction, PM_>4_), quantitatively recovered from the interior surfaces of the cyclone.

A comparison between concentrations of fosetyl (ng·m^−3^) in the inhalable and respirable fractions was evaluated. The concentrations in ng·m^−3^ were obtained taking into account the total volume of the air sampled on each filter of about 0.4 m^3^, and the results are expressed as *x*_*m*_ ± standard deviation (*σ*) of the method. Fosetyl in the inhalable and respirable fractions of PM, sampled inside the tractor, was not detected, whereas outside the cabin, the concentration of fosetyl was 246.3 ± 2.5 ng·m^−3^ in PM_4_ and 2900.0 ± 150 ng·m^−3^ in PM_>4_.

The higher fosetyl concentration in PM_>4_ was expected since the spray mixture was dispensed as emulsion, and the relative particles have a large aerodynamic diameter. The results show that the operator inside the tractor cabin is safe, whereas possible workers nearby could be exposed. To our knowledge, in the literature, no other data about this topic are present to be compared to the obtained results.

## 4. Conclusions

Several directives and guidelines set maximum levels of pesticides in water in order to protect the human and environmental health [20]. In the European Union, the MRLs for pesticide residues in food are established, for example, fosetyl limit in grapes is 100 mg·kg^−1^ [21]. To date, no limits have been established on airborne pesticides; few data on their concentrations in the respirable or inhalable fractions of the particulate matter are present in the literature [19, 22].

EPA method for the analysis of fosetyl-Al in food is quite complicated in terms of purification and analysis [23]; hence, to measure fosetyl low concentration in PM_4_, during the spreading operation, we proposed a highly selective, sensitive, and accurate method based on liquid chromatography-tandem mass spectrometry. Due to the highly polar nature of the compound, it was mandatory to use a column based on hydrophilic interaction that allowed to have a proper retention time.

The method gave good results in terms of recovery, linearity, LOD, LOQ, precision, and accuracy [24].

Finally, it was applied to environmental samples collected inside/outside a tractor cabin during a spreading campaign on the agricultural field. The samples were prepared using water, both as extracting and eluting solvent by cartridges. No matrix effect was found. The results highlighted that the cabin protects the operator from the penetration of the pesticides. On the other hand, the presence of pesticides in PM_4_ and especially in PM_>4_ outside the cabin would demonstrate that a possible exposure might affect the people working or living next to the pesticide application site. The proposed methodology provides a useful tool for research purposes, for epidemiological and risk assessments, in order to plan eventual action strategies.

## Figures and Tables

**Table 1:** Operating parameters in MS and MS/MS experiments (infusion (10 *µ*L·min^−1^) of fosetyl aqueous solution at 10 mg·L^−1^).

Parameters	Value
Gas temperature (°C)	300
Gas flow (L/min)	5
Nebulizer (psi)	60
Sheath gas temperature (°C)	400
Sheath gas flow (L/min)	11
Capillary voltage (V)	2000
Nozzle voltage (V)	500
Fragmentor (V)	70
Collision energy (eV)	20

**Table 2:** LOD/LOQ values for fosetyl expressed as ng·mL^−1^, pg·m^−3^ (sampled air volume of 0.4 m^3^), and mg·kg^−1^ of sampled particulate matter.

	LOD			LOQ		
	ng·mL^−1^	pg·m^−3^	mg·kg^−1^	ng·mL^−1^	pg·m^−3^	mg·kg^−1^
Fosetyl	0.3	75	0.5	1.0	250	1.7

**Table 3:** Parameters of the current method compared to those obtained by other authors in food.

Parameter	Current method	Reference [9]	Reference [18]
LOD	0.3 ng·mL^−1^	50 ng·mL^−1^	0.1–0.2 ng·mL^−1^
Accuracy	>90%	98–106%	nr
Precision	<6%	10%	<12%
Recovery	>80%	98–106%	50–200%
Matrix effect	<10%	>10%	<10%

nr: not reported.
